# A Multimodal Approach to Identify Metallothionein Metal Inducers in Nile Tilapia: Insights From Molecular Docking and Hepatocyte Exposure

**DOI:** 10.1002/jat.4914

**Published:** 2025-09-02

**Authors:** Jessica Zablocki da Luz, Tugstênio Lima de Souza, Aliciane de Almeida Roque, Micheli de Marchi, Roberta Pozzan, Camila Confortin, Iracema Opuskevitch, Fernando Cesar Alves da Silva Ferreira, Ciro Alberto de Oliveira Ribeiro, Francisco Filipak Neto

**Affiliations:** ^1^ Grupo de Pesquisa em Toxicologia Celular, Departamento de Biologia Celular Universidade Federal do Paraná Curitiba PR Brazil; ^2^ Copel GeT‐SOS/DNGT Curitiba PR Brazil

**Keywords:** Cadmium, Hepatocyte primary culture, Metallothionein, Molecular docking, Nile tilapia

## Abstract

Many human activities contribute to the pollution of aquatic ecosystems, primarily through agricultural, industrial, mining, and domestic discharges into water bodies. Fish, being highly sensitive to environmental changes, serve as valuable models for monitoring the health of these ecosystems. Metallothionein (Mt), a biomarker for metal contamination, shows variable expression depending on the metal involved. Transcription of the *mt* gene is regulated by intracellular metal concentrations and mediated by interactions between metal‐responsive transcription factors (Mtf) and metal response elements (MRE) in the *mt* promoter. Zinc plays a key role by binding to Mtf, activating it, and enabling interaction with MRE sequences to initiate transcription. In this context, this study aimed to identify the most potent inducers of *mt* expression in *Oreochromis niloticus* . Initially, zinc‐binding proteins from *O. niloticus* were modeled to assess differential binding scores of various metals using molecular docking, which suggested the potency ranking Cd^2+^ > Cu^2+^ > Mn^2+^ > Hg^2+^ > Pb^2+^. These predictions were validated using primary hepatocytes exposed to concentrations 10 times higher than the maximum allowed for effluent discharge under Brazilian law. The expression of both *mt* mRNA and Mt protein was evaluated in hepatocytes. Both *in silico* and in vitro results identified cadmium as the most potent inducer of Mt. Other metals did not induce *mt* expression under the tested conditions. These findings underscore the importance of understanding how Mt expression varies by metal and tissue, as differences in responsiveness can influence the interpretation of Mt levels in teleost fish used for water quality monitoring and environmental toxicology.

## Abstract 2

Human activities discharge toxic metals into aquatic ecosystems, threatening fish, key indicators of environmental health. Metallothionein (Mt) gene expression, regulated by metal transcription factors (Mtf) and metal regulatory elements (MRE), serves as a biomarker of metal exposure. This study identified potent inducers of Mt in *Oreochromis niloticus* using molecular docking and in vitro assays. Cadmium (Cd^2+^) emerged as the strongest inducer (Cd^2+^ > Cu^2+^ > Mn^2+^ > Hg^2+^ > Pb^2+^), with other metals failing to induce Mt expression in primary liver cells.

## 1 Introduction

Metals can become toxic to aquatic life when their concentrations exceed certain thresholds (Tchounwou et al. 2012). Primary natural sources of metals include the erosion and chemical leaching of soils and rocks, with secondary contributions from the decomposition of organic matter (Tchounwou et al. 2012). However, human activities, including industrial operations, mining, agriculture, and domestic waste disposal, have significantly elevated the concentrations of certain metals in these ecosystems (Mateo‐Sagasta et al. 2017). These activities are responsible for the discharge of millions of tons of metals into water bodies (Mateo‐Sagasta et al. 2017). Currently, the global human population is about 8 billion, and projections suggest that it can reach 8.5 billion in 2030, 9.7 billion in 2050, and 10.4 billion in 2100 (UN 2022). As human activities and waste production continue to grow, freshwater ecosystems face increasing risks from metals and other pollutants, given their role as the ultimate recipients of many contaminants (Vaghela et al. 2017).

In particular, metallothionein (Mt) is a protein that responds primarily to stress caused by metals in organisms, such as fish (Viarengo et al. 1999). Mt is a small cysteine‐rich protein, first detected in the equine renal cortex, which binds and exchanges specific metal ions (Margoshes and Vallee 1957). After metallothionein's first detection and isolation, it took almost 20 years to be identified in teleosts. Since then, many studies have focused on the use of Mt as a biomarker of aquatic metal pollution (Olsson 1996). Due to its ability to bind metals, respond to trace elements, and show tissue‐specific expression, it serves as a useful biomarker for environmental monitoring and may also aid in water treatment (R. Yang et al. 2024). Mt levels in aquatic organisms have been widely used to assess metal bioavailability and exposure in freshwater and marine environments, aiding in the evaluation of ecosystem health and supporting regulatory frameworks for pollution control (R. Yang et al. 2024).

The ability of different metals to trigger a response in Mt expression varies considerably, with some metals being more potent inducers than others (Atli and Canli 2008). The transcription of the *mt* gene depends on the intracellular concentration of metals, and it is controlled by interactions between metal transcription factors (Mtf) and metal regulatory elements (MRE). Zinc (Zn) plays a key role by binding to and activating Mtf, which interacts with the MRE sequences of the Mt promoter, leading to *mt* gene transcription (Dong et al. 2015; Günther et al. 2012). Other metals do not act directly on transcription factors, but displace Zn from many proteins, leading to an increase in intracellular Zn concentration and leaving it free to interact with Mtf (Dong et al. 2015). However, different animal species may present minor peculiarities in this general mechanism. For example, compared to mouse, Mt fish have greater Zn mobility, leading to differences in reactivity (Capasso et al. 2003).

The evaluation of metal concentrations through chemical analysis of the water may not accurately reflect their potential toxicity in aquatic ecosystems, so that further analysis involving the biota is necessary to determine the safe levels of waste that can be discharged into water bodies (Bolis et al. 2001). Analyzing biomarkers in biota is a fundamental approach for monitoring the toxic effects of environmental contaminants and pollutants (Strimbu and Tavel 2010). At the molecular level, RNA expression, protein and metabolic profiling (Hariharan et al. 2016) can be used to assess the risk of environmental exposure (Livingstone 1993).

Different approaches can be applied to investigate how metals interfere with Mt expression, such as in silico, in vitro, and in vivo approaches, and, therefore, contribute to a better interpretation of results of Mt detection (Atli and Canli 2008; Chan et al. 2006; Lu et al. 2022). Although MT can be expressed in other organs, the liver is crucial when it comes to metal detoxification (Chatterjee et al. 2016). Cell lines and primary hepatocytes are the main in vitro models for testing liver toxicity, each with its own advantages and disadvantages. Primary hepatocytes are particularly useful since they are more sensitive and could provide more environmentally relevant responses (Soldatow et al. 2013).

To identify which metals most strongly induce metallothionein (Mt) production in *Oreochromis niloticus* (Nile tilapia), a key step toward interpreting Mt levels in biomonitoring studies using this species, first, this study employed molecular docking techniques. By analyzing the interactions between various metals and Zn‐binding protein structures, binding affinities were compared to predict the most potent Mt inducers. Then, primary hepatocytes from *O. niloticus* were used to validate the results generated in the *in silico* approach, using exposure concentrations 10 times higher than the maximum value allowed for effluent discharge according to Brazilian law (CONAMA 2008) to assess *mt* expression.

## 2 Material and Methods

### 2.1 Molecular Docking

To begin, functional interaction networks were generated using the STRING tool (Szklarczyk et al. 2020), selecting metal transcription factor 1 (Mtf1) as the input protein and *O. niloticus* as the reference organism. Next, potential zinc‐binding proteins were identified through the PredZinc server (Shu et al. 2008), which predicts metal‐binding residues. Proteins identified as zinc binders were then submitted for molecular docking via the MIB2 server, using structural models obtained through homology modeling with PS2 (Lu et al. 2022). Among the docking results, the interaction showing the strongest affinity with Zn^2+^ was selected, and comparable binding scores for other metals were examined at binding sites containing at least one conserved residue. Binding site visualization and structural analysis were performed using PyMOL (Version 3.0).

### 2.2 Primary Hepatocyte Culture

*O. niloticus* fish were obtained from a fish farming station and kept in a tank with filtered tap water, constant aeration, and fed every 2 days. For cell isolation, the fish were anesthetized with MS‐222 (ethyl 3‐aminobenzoate methanesulfonate, 0.02% in water) and killed by spinal cord section. All procedures using the animals were approved by the *Animal Use Ethics Committee of the Biological Sciences Sector of the Federal University of Paraná* (CEUA/BIO – UFPR) with certificate number 1286.

Liver cells isolation and culture procedures were performed as previously described in Zablocki da Luz et al. (2024). The primary culture of liver cells predominantly comprises hepatocytes, which constitute approximately 70% of the total liver cell population (Gao et al. 2008). Therefore, this will be referred to as a hepatocyte culture. For the viability assays, hepatocytes were cultured in 96‐well cell culture plates at a density of 2.1 × 10^5^ cells cm^2^. For the Mt indirect detection assays, hepatocytes were cultured in 6‐well plates at a density of 2.1 × 10^5^ cells cm^2^. For RT‐qPCR assay, hepatocytes were cultured in 6‐well plates at a density of 3.1 × 10^5^ cells cm^2^. For immunocytochemistry assay, hepatocytes were cultured in 24‐well plates with round glass coverslips at the bottom at a density of 3.1 × 10^5^ cells cm^2^.

### 2.3 Cell Viability Assay

Cells were exposed to five environmentally relevant concentrations of cadmium chloride, CdCl_2_ (0.02; 0.04; 0.2; 1 and 2 mg L^−1^). After 24, 48, and 72 h exposure, MTT metabolism, neutral red uptake, and cell attachment assays were performed. Cell viability was also assessed for hepatocytes exposed for 72 ho other metals in concentrations 10 times higher than the maximum value allowed for effluent discharge according to Brazilian law (CONAMA 2008): lead (5 mg L^−1^, Pb (OOCCH_3_)), copper (10 mg L^−1^, CuCl), manganese (10 mg L^−1^, MnCl_2_), and mercury (0.1 mg L^−1^, HgCl_2_). MTT metabolism, neutral red uptake, cell attachment, and resazurin metabolism were evaluated with the modifications described in Zablocki da Luz et al. (2024). Control cells were kept in culture under the same conditions as metal‐exposed cells, except for the absence of the metals.

#### 2.3.1 Metallothionein Induction—mRNA Level

The expression of the *mt* gene was evaluated in hepatocytes exposed to 2 mg L^−1^ cadmium, 5 mg L^−1^ lead, 10 mg L^−1^ copper, 10 mg L^−1^ manganese, and 0.1 mg L^−1^ mercury. Also, the expression of the *mt* gene was evaluated in cells exposed to a lower concentration of cadmium (1 mg L^−1^), which did not present high toxicity like the previous one, and gonadotropin‐releasing hormone agonist (GnRH‐A, 100 nM), which showed an inhibitory effect on zinc‐induced Mt mRNA production in HepG2 and HuH7 cells (Pati et al. 1996). After 72 h exposure, hepatocytes were harvested from 2 wells of the 6‐well cell culture plates per experimental group.

First, cell scrapers were used to detach the cells from the culture plates. Second, cell suspension was transferred to a 2 mL microtube and centrifuged at 50 *g* for 3 min at room temperature. After removing the supernatant, 100 μL of RNAlater (Invitrogen, ref. AM7021) was added to the samples. The PureLink RNA Mini Kit (Invitrogen, ref. 12183018A) was used for RNA extraction with an additional step using the Trizol reagent (Invitrogen, ref. 15596026). To remove possible contamination with genomic DNA, TURBO DNA‐free (Invitrogen, ref. AM1907) was used following manufacturer recommendations. To quantify the extracted RNA, Quantifluor (Promega, ref. E2671) was used following manufacturer recommendations. Additionally, the RNA quality assessment was performed to check purity, using the Abs_260_/Abs_280_ ratio for protein contamination and Abs_260_/Abs_230_ for phenol and other contaminants, and RNA integrity was confirmed using agarose gel electrophoresis. Then, the synthesis of cDNA from mRNA was performed using the SuperScript IV First‐Strand Synthesis System (Invitrogen, ref. 18091050) and Veriti Thermal Cycler. A total of 10 ng of cDNA and 500 nM of primers were used per reaction in 10 μL volume. For real‐time amplification, PowerUp SYBR Green Master Mix (Invitrogen, ref. A25776) and the StepOnePlus Real‐Time PCR System were used. For the analysis, the Web‐based LinRegPCR server was used (Untergasser et al. 2021).

The reference mRNAs used were ubiquitin‐conjugating enzyme (*ubce*), tubulin alpha chain‐like (*tuba*), and beta actin (*actb*) (C. G. Yang et al. 2013). The target mRNA was metallothionein (*mt*) (Zhang et al. 2022) (Table 1). Three independent experiments were performed.

**TABLE 1:** Primer information.

Genes	Abbr.	Sequence (5′‐3′)	GenBank accession number	Amplicon (bp)
Ubiquitin‐conjugating enzyme	*ubce*	F: CTCTCAAATCAATGCCACTTCC	XM_003460024	130
		R: CCCTGGTGGAGGTTCCTTGT		
Tubulin alpha chain‐like	*tuba*	F: AGCCAGACGGACAGATGCC	XM_003445344	153
		R: TTCCTGCACGCACCTCATC		
Beta actin	*actb*	F: GTACCCCATTGAGCACGGTA	XM_003455949	122
		R: GAGCCTCTGTGAGCAGGACT		
Metallothionein	*mt*	F: AAGAGCCACTCCTACACCGT	XM_003447045.5	139
		R: TTGCAGGTTCCAGTCTTGGC		

#### 2.3.2 Protein Level

To compare Mt protein levels between the experimental groups exposed to four different concentrations of Cd (0.02; 0.04, 0.2, and 1 mg L^−1^), a colorimetric assay was used with modifications (Viarengo et al. 1997). After 48 and 72 h exposure, hepatocytes were collected from two wells of six‐well cell culture plates per experimental group. First, the cells were detached using cell scrapers, the cell suspension was transferred to a 2 mL microtube, and centrifuged at 50 *g* for 3 min at room temperature. Pelleted cells were homogenized in 20 mM Tris–HCl buffer, pH 8.6 using 3 mm metal beads and TissueLyser II homogenizer (Qiagen), followed by centrifugation (15,000 *g* for 30 min, 4°C). Total proteins in the supernatant were quantified through the Bradford assay (Bradford 1976) using a dye reagent (Bio‐rad, ref. 5000006). Then, 300 μL of the supernatant were transferred to a new tube and treated with 342 μL of ethanol/chloroform (13.3:1). The samples were centrifuged (6000 *g* for 10 min, 4°C), 490 μL of the supernatant was transferred to a new tube, and 1.502 mL of ethanol/HCl (45:1) was added. The samples were kept at −20°C for 1 h and centrifuged again (6000 *g* for 10 min, 4°C). The supernatant was removed and 1 mL of chloroform/ethanol/20mM‐Tris–HCl (1:87:12) was added. After new centrifugation, the supernatant was removed, 50 μL of 250 mM NaCl solution and 50 μL of 1 M HCl containing EDTA 4 mM were added, and the samples were vortex‐mixed. Then, 1 mL of 0.43 mM DTNB ((5,5′‐dithiobis‐(2‐nitrobenzoic acid)), Sigma‐Aldrich, ref. D8130) dissolved in methanol and then in 0.2 M Na‐phosphate buffer was added, and samples were centrifuged (3000 *g* for 5 min). The supernatant was added to the 96 well microplates, as well as a glutathione, GSH (Vetec, ref. 1843) reference curve, and the absorbance was measured at 412 nm. Considering that Mt has between 20% and 30% of cysteine, the Mt content was estimated, equal to 0.3 times SH content. Four independent experiments were performed.

#### 2.3.3 Immunocytochemistry

Hepatocytes were exposed to 1 mg L^−1^ of Cd for 72 h and then fixed in 4% paraformaldehyde (Sigma‐Aldrich, ref. 158127) in phosphate buffer saline (PBS) for 30 min, permeabilized with 0.2% Triton‐X in PBS for 10 min, and rinsed once with PBS for 5 min. To block nonspecific sites, the cells were incubated with 1% bovine serum albumin (BSA) and 22.52 mg mL^−1^ of glycine in PBS‐T (PBS + 0.1% Tween 20) for 30 min. For the detection of the targets (Mt and Mtf), the cells were incubated with the primary antibodies anti‐FTM (ABCAM, ref. AB183897) and anti‐Mt (Nagamatsu et al. 2020) diluted 1:200 in 1% BSA in PBS‐T for 1 h at room temperature. The cells were then rinsed three times in PBS for 5 min, incubated with Cy 3‐conjugated secondary antibody (1:150, Jackson, ref. 305166047) in 1% BSA for 1 h at room temperature in the dark, and rinsed again three more times. For nucleus and actin cytoskeleton labeling, the cells were incubated with 5 μg mL^−1^ of 4′,6‐diamidino‐2‐phenylindole (DAPI, Sigma‐Aldrich, ref. D1306) for 1 min and with phalloidin‐FITC (66 μM, Invitrogen, ref. F432) for 20 min. The coverslips were mounted in fluoromount (EMS, ref. 17984) and images were captured on a confocal microscope (Nikon Eclipse Ti microscope, Japan).

### 2.4 Statistical Procedures

Means of the technical replicates of each experimental group were used. The data presented are equivalent to four independent experiments, which were normalized compared to the control group. For analysis, the data were first tested for the homoscedasticity of the variances and normality of the residuals, using the Bartlett and Shapiro–Wilk tests, respectively. One‐way or two‐way ANOVA was performed, followed by Fisher's *post hoc* test to compare the groups. A *p* < 0.05 was considered statistically significant.

## 3 Results

### 3.1 Cell Viability Assay

**FIGURE 1:**
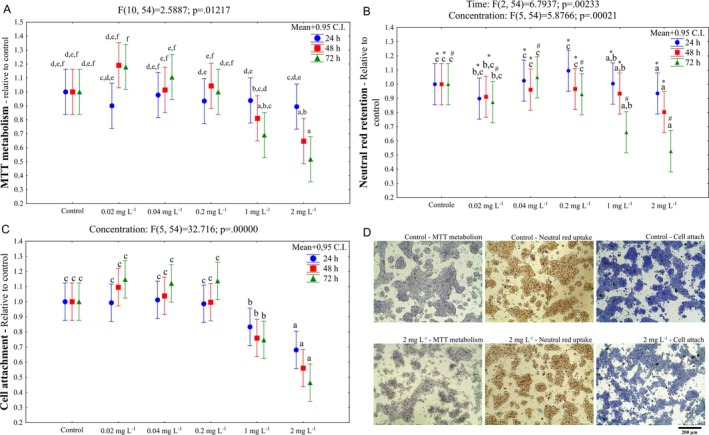
Cell viability of *Oreochromis niloticus* hepatocytes exposed to CdCl_2_. Neutral red retention (A), MTT metabolism (B), and cell attachment (C) assays after 24, 48, and 72 h‐exposure to CdCl_2_. Images (D) were captured under an inverted light microscope (Leica Microsystems, Germany) after 72 h‐exposure and exemplify culture fields right before dye extraction for quantification (results plotted in A, B, and C) through absorbance measurement in spectrophotometer. Letters indicate differences among CdCl_2_ concentrations and symbols (* and #) indicate differences among exposure times when the interaction between the factors was not observed. Mean +0.95 C.I. Bar = 200 μm.

A decrease in cell viability was observed in the cells exposed to 1 and 2 mg L^−1^ of CdCl_2_ (Figure 1): MTT metabolism assay (decreases between 30% and 50%, Figure 1A), neutral red retention assay (decreases between 10% and 45%, Figure 1B) and cell attachment assay (decreases between 15% and 55%, Figure 1C). Effects were usually harsher in the cells exposed to 2 mg L^−1^ of CdCl_2_ for 72 h.

**FIGURE 2:**
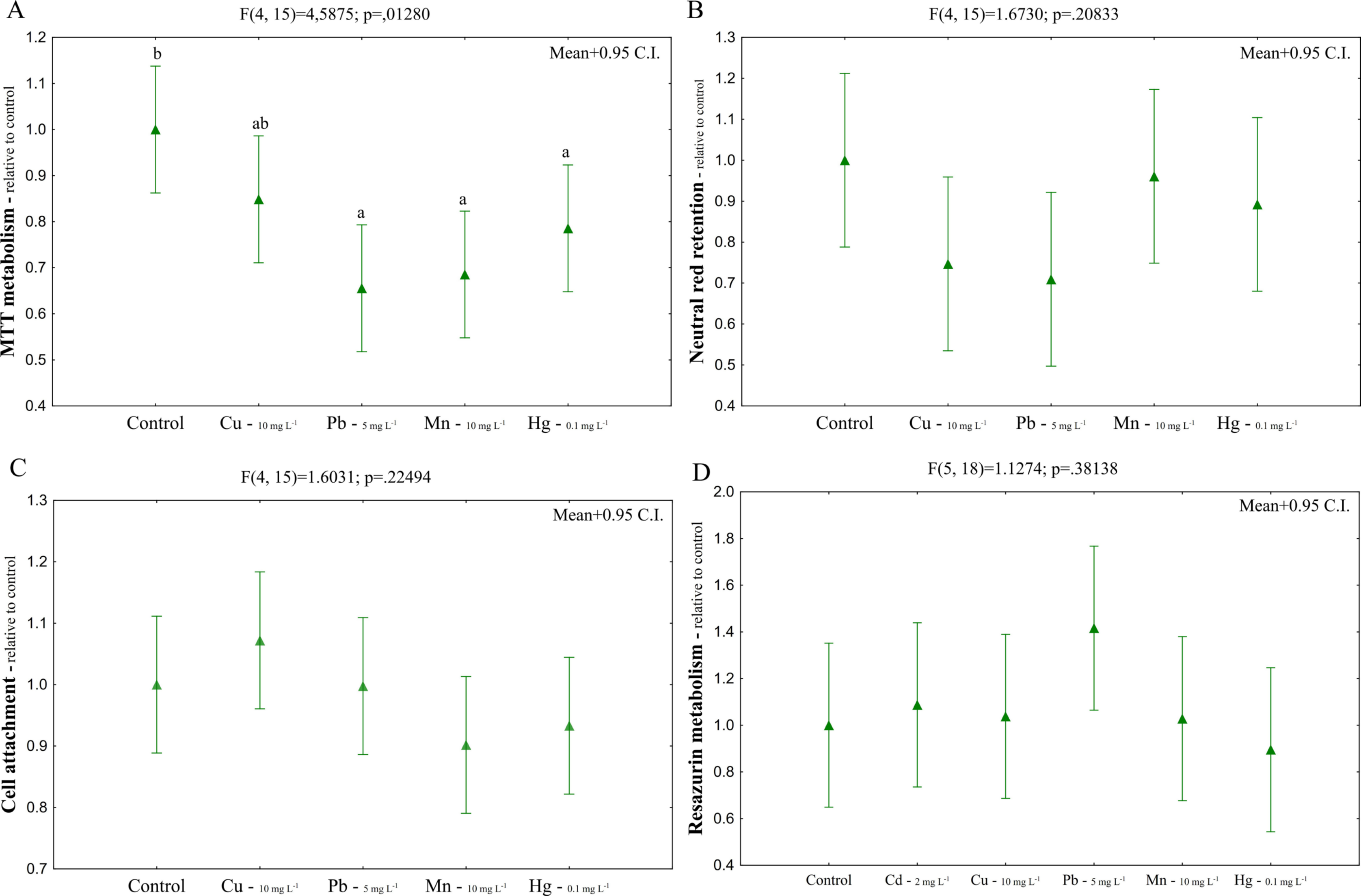
Cell viability of *Oreochromis niloticus* hepatocytes exposed to CuCl, Pb (OOCCH_3_), MnCl_2_ and HgCl_2_. MTT metabolism (A), Neutral red retention (B), Cell attachment (C) and Resazurin metabolism (D) assays after 72 h‐exposure. For Resazurin metabolism assay, CdCl_2_ was also tested (2 mg L^−1^). Letters indicate differences among used metal concentrations in relation to the control group. Mean+0.95 C.I.

For the other metals, significant effects in cell viability were observed only for the MTT metabolism, with decreases of ~35% for lead, ~30% for manganese, and ~25% for mercury (Figure 2A–D). Since the concentrations used did not result in significant changes in cell viability parameters, such as neutral red retention, cell attachment, and resazurin metabolism, after 72 h exposure, and only a reduction in MTT metabolism was observed for Pb, Mn, and Hg, this exposure time was also used in the subsequent analyses.

### 3.2 Molecular Docking and mt Gene Expression

**FIGURE 3:**
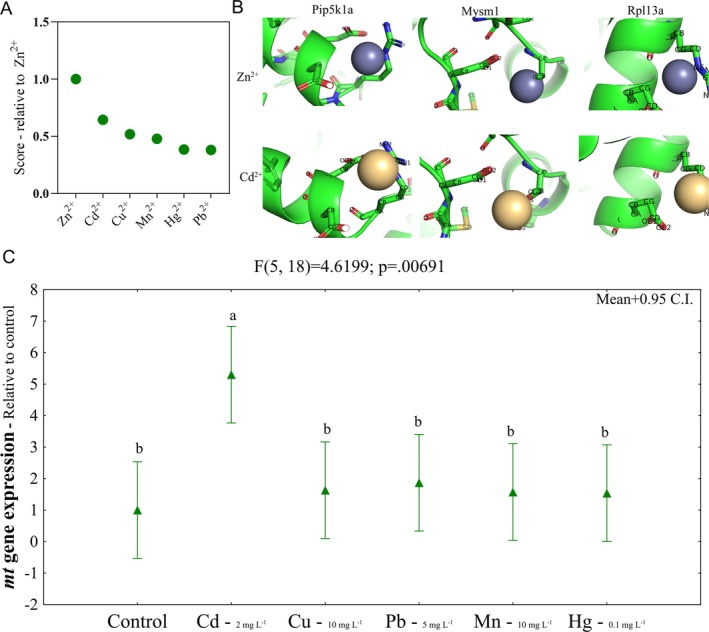
Molecular docking between Zn^2+^, Cd^2+^, Cu^2+^, Hg^2+^, Mn^2+^, and Pb^2+^ and Zn‐binding proteins. Cd^2+^ had the highest score, after Zn, compared to the other metals (A). Some of the proteins with high score for Cd^2+^ are represented (B). RT‐qPCR for *Oreochromis niloticus* hepatocytes exposed to 2 mg L^−1^ of CdCl_2_, 10 mg L^−1^ of CuCl, 5 mg L^−1^ of Pb (OOCCH_3_), 10 mg L^−1^ of MnCl_2_ and 0.1 mg L^−1^ of HgCl_2_.

A total of 200 proteins that form functional interaction networks were used to search for those with Zn‐binding sites. From these, 38 proteins with Zn‐binding sites were identified and selected for molecular docking with the metals Zn^2+^, Cd^2+^, Cu^2+^, Mn^2+^, Hg^2+^, and Pb^2+^. Compared to Zn (mean score = 100%), the mean percentage scores for Zn‐binding sites were 38.08%, 38.40%, 47.80%, 51.86%, and 64.49% for Pb^2+^, Hg^2+^, Mn^2+^, Cu^2+^, and Cd^2+^, respectively (Figure 3A). The scores are available in Data S1. Pip5k1a, Mysm1, and Rpl13a are some of the proteins with high scores for Cd^2+^ binding in Zn‐binding sites (Figure 3B). The expression of *mt* was ~400% higher in cells exposed to CdCl_2_ than in the control (Figure 3C). No differences in *mt* expression were detected in hepatocytes exposed to the other metals at environmentally relevant concentrations (Figure 3C). Fold change values corresponding to the results are available in Data S2.

### 3.3 Metallothionein Induction by Cd

**FIGURE 4:**
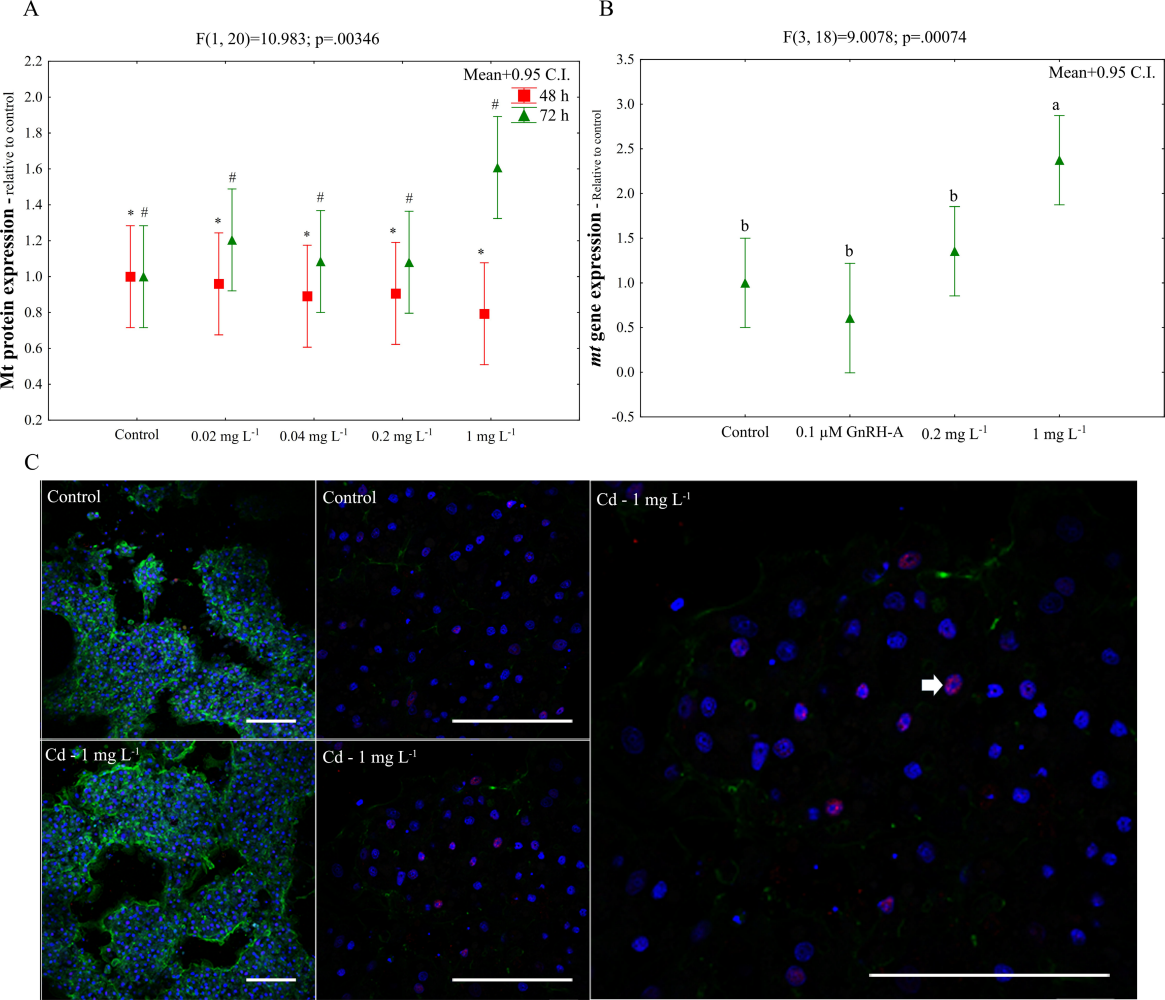
Metallothionein induction in *Oreochromis niloticus* hepatocytes exposed to CdCl_2_. Mt protein content (A), RT‐qPCR (B) and immunocytochemistry (C) assays. Letters indicate differences among concentrations and symbols (* and #) indicate differences among exposure time points when the interaction between the factors was not observed. Blue: cell nucleus labeled with DAPI. Green: actin cytoskeleton labeled with falloidin‐FITC. Red: Mtf detected with Cy 3‐conjugated secondary antibody. White arrow: Mtf labeling. Mean+0.95 C.I. Bar = 100 μm.

For the protein level, ~30% increase in Mt content was observed in hepatocytes exposed to Cd for 72 h compared to 48 h (Figure 4A). For mRNA level, ~150% increase in *mt* content was observed in the cells exposed to 1 mg L^−1^ Cd for 72 h (Figure 4B), but there was no difference for those exposed to 0.1 μM GnRH‐A. Mtf labeling was visualized by immunocytochemistry (Figure 4C).

## 4 Discussion

The study of metal‐binding sites in proteins, such as Mt, is important to understand the cell processes in which proteins are involved (Waldron et al. 2009). However, it can be challenging experimentally considering the number of complicated steps or specialized techniques, such as metal‐affinity column chromatography and electrophoretic mobility shift assays, nuclear magnetic resonance spectroscopy, absorption spectroscopy, and gel electrophoresis (Lin et al. 2016; Lu et al. 2022). Computational biology can be used to investigate the possible interaction between toxic metals and health risks to organisms (Gupta et al. 2020) and in contrast, facilitates efficient identification and evaluation of metal‐binding regions (Lin et al. 2016; Lu et al. 2022). MIB2, e.g., is a server for modeling and predicting binding sites for metal ions, based on the fragment transformation method (Lu et al. 2022). In the present study, cadmium exhibited the highest binding score mean for Zn‐binding sites among the tested metals (Cd^2+^ > Cu^2+^ > Mn^2+^ > Hg^2+^ > Pb^2+^), as predicted by the MIB2 server. This in silico result supports the known ability of the compound to displace Zn^2+^ and activate metal‐responsive pathways. Although zinc was included in the molecular docking analysis as a physiological reference, given its natural affinity for Mtf, it was excluded from the in vitro validation. This decision was based on several considerations. The maximum allowable concentration for zinc in effluent discharge, according to Brazilian legislation (CONAMA 2008), is relatively high compared to other metals (5.0 mg L^−1^), underscoring its distinct regulatory status. Moreover, while zinc is an essential element required for numerous structural and enzymatic functions, it can become harmful when present at excessively high or low concentrations. As noted by Castaldo et al. (2021), the delayed accumulation of Zn in organisms reflects the efficiency of homeostatic mechanisms that tightly regulate its levels; however, its basal presence in culture media may interfere with experimental treatments and obscure the specific effects of exogenously added Zn^2+^, thereby complicating the interpretation of changes in Mt expression.

Consistently, in vitro exposure of hepatocytes to CdCl_2_ for 72 h resulted in a marked, dose‐dependent upregulation of *mt* mRNA levels: approximately 150% and 400% increases were observed at concentrations of 1 and 2 mg L^−1^, respectively. These findings reinforce the predictive value of computational modeling for identifying potential Mt inducers and demonstrate that cadmium's high predicted affinity translates into strong transcriptional activation of *mt* in exposed cells. The well‐established role of cadmium as a potent Mt inducer further corroborates our observations (Atli and Canli 2008; Chouchene et al. 2022; Nursanti et al. 2017). For instance, the controlled study by Atli and Canli (2008) exposed *O. niloticus* to 0, 5, 10, and 20 μM of Cd^2+^, Cu^2+^, Zn^2+^, and Pb^2+^ for 14 days. The results showed that although lead accumulated in liver and gill, only cadmium exposure led to a significant increase in hepatic Mt levels; Pb did not elicit an Mt response under those parameters. This outcome aligns with our findings and suggests metal‐specific induction thresholds and affinitive dynamics. A similar increase was reported in different studies for different fish species, such as *Danio rerio* and *Ctenopharyngodon idella* (Jabeen et al. 2022; Shen et al. 2019; Zhu et al. 2018). Given these results, the expression of Mt is likely indicative of elevated Cd levels, as other elements at concentrations exceeding Brazilian legal limits did not trigger its expression. Although other studies have identified several metals as potential inducers of Mt in *O. niloticus* , the experimental models and approaches used differ significantly from those applied in the present study. For example, Cheung et al. (2004) reported that all tested metal ions (Cu^2+^, Cd^2+^, Hg^2+^, Ni^2+^, Pb^2+^, and Zn^2+^) induced hepatic *mt* mRNA expression. However, their study involved intraperitoneal injections of single doses (1, 5, and 10 mg kg^−1^), with samples analyzed with 24 h exposure. Also, it is important to emphasize some experimental limitations of the present study: (1) the in vitro model may not fully replicate in vivo physiology, since systemic factors such as immune, endocrine, or whole‐body interactions that modulate Mt expression are absent; (2) temporal and concentration constraints may have limited the induction of metals such as Pb, as longer exposures, alternative concentrations, or different timing might be required to elicit a detectable response; and (3) the sensitivity of detection methods could have prevented the observation of modest increases in Mt expression, which might otherwise become evident in in vivo systems where systemic interactions could enhance transcriptional responses.

For protein level, we observed an increase of ~30% in Mt content in the cells exposed to 1 mg L^−1^ of CdCl_2_ for 72 h compared to 48 h. Mt levels were indirectly estimated by detecting sulfhydryl groups using Ellman's reagent and measuring absorbance at 412 nm, taking advantage of the unusually high cysteine content (20%–30%) in these proteins relative to others in ethanolic extracts (Viarengo et al. 1997). Zhu et al. (2018) also used the indirect method to quantify the content of Mt in the liver of female zebrafish exposed to CdCl_2_ for 15 weeks and observed an increase of ~100% and ~300% of Mt content for 2.5 and 5 μg L^−1^ of CdCl_2_ concentrations, respectively.

Interactions between proteins and specific metal ions are essential processes in many biochemical and physiological processes (Foster et al. 2022; Grüngreiff et al. 2016). Metals are required in specific proteins to impart structure, e.g., zinc fingers, or to assist catalysis by acting as co‐factors in enzymes (Foster et al. 2022). Half of the enzymes structurally characterized by experiments in the protein data bank need metals (Waldron et al. 2009). Metal‐binding sites compete for limited amounts of exchangeable metals in normal biological conditions (Foster et al. 2022). So, protein binding of an inappropriate metal that can change the structure is not common (Foster et al. 2022).

Zinc is an essential element, critical for a large number of structural proteins, enzymatic processes, and transcription factors, and plays fundamental roles in cell metabolism, division, growth, and differentiation (Grüngreiff et al. 2016). Zinc also mediates intracellular metal ion balance (Dong et al. 2015). Changes in intracellular concentration of Zn^2+^ influence the structure of metal response transcription factor 1 (Mtf‐1), which is activated by the binding of Zn^2+^ to the six zinc fingers, a DNA‐binding domain involved in metal regulation (Dong et al. 2015; Günther et al. 2012). The activation of Mtf‐1 induces the expression of the metallothionein gene (Dong et al. 2015). Other metals do not act directly on Mtf‐1, but as they can bind to other Zn‐binding proteins, they can replace this metal, provoking an increase in intracellular Zn^2+^ concentration (Roesijadi 1992).

In the present study, functions of some identified Zn‐binding proteins, with which cadmium had a great affinity, include response to stress, biosynthetic process, transport, metabolic process, and catalytic activity. Consequently, Cd can dysregulate those functions through the metabolic disturbances that may contribute to Cd toxicity. F. Hu et al. (2021) and W. Hu et al. (2022) showed that environmentally relevant levels of Cd induce adaptive responses with compensatory mechanisms in *D. rerio* and *O. niloticus* , which may help to maintain fish survival at the cost of growth. W. Hu et al. (2022) suggest that increases of Mts, heat shock protein 70, and the expression of genes related to ribosome, protein processing in the endoplasmic reticulum, and protein export pathways may be compensatory responses following oxidative stress, endoplasmic reticulum stress, and apoptosis after Cd exposure.

Also, in this study, we used more than one assay to assess cell viability. A multiparametric approach enhanced reliability and the interpretation of subtle versus generalized toxic effects (Fotakis and Timbrell 2006). The combination of MTT, resazurin, neutral red uptake, and cell attachment assays provided complementary insights into mitochondrial activity, metabolic capacity, lysosomal integrity, and cell attachment. In cells exposed to 2 mg L^−1^ of CdCl_2_, consistent reductions across all assays indicated generalized cytotoxicity, particularly at 72 h exposure. In contrast, Pb, Mn, and Hg induced moderate viability loss detectable only by the MTT assay (~25%–35% reduction), suggesting selective mitochondrial impairment without broader damage.

Environmental toxicology, which involves evaluating chemical toxicity and understanding how chemicals contribute to disease, has seen significant advancements in recent years (Stegeman et al. 2010). The integration of molecular biology into environmental toxicology has become more popular in recent decades, and cell and physiology research on molecular mechanisms of chemical action has provided fundamental insights into basic control processes (Stegeman et al. 2010). In this context, the importance of monitoring water quality and research in environmental toxicology using biomarkers, such as metallothionein for metal contamination/pollution, has been recognized (Kadim and Risjani 2022). In this study, both in silico and in vitro approaches were employed. As fish are highly sensitive to environmental changes, they are a good model for monitoring aquatic ecosystems, as they metabolize, detoxify, and accumulate toxic chemicals (Shahjahan et al. 2022). *O. niloticus* is an interesting experimental model since it is easy to maintain in high density, has rapid growth, and is resistant to many diseases (Vicente et al. 2014).

Finally, understanding how biomarkers for metal contamination are expressed in different tissues (Shahjahan et al. 2022; Shen et al. 2019), the difference in responsiveness to exposure to different metals (Chan et al. 2006; Chatterjee et al. 2016; Nursanti et al. 2017; Olsson 1996; Yudkovski et al. 2008; Zhang et al. 2022), and how other chemicals (Bauman et al. 1991; JBauman et al. 1992a, 1992b; Carvalho et al. 2012; Ceyhun et al. 2012; Erdoğan et al. 2011; Mosleh et al. 2005a; Mosleh et al. 2005b; Mosleh et al. 2003) can interfere is important for interpreting Mt levels found in teleost fish in water quality monitoring and environmental toxicology research.

## 5 Final Comments

Cadmium was identified as the most effective inducer of metallothionein expression in *O. niloticus* hepatocytes, based on both in vitro and in silico analyses, while other metals did not induce *mt* expression under the tested conditions. This response may reflect cadmium's strong affinity for zinc‐binding proteins, which increases free intracellular zinc and indirectly enhances *mt* transcription. These findings highlight the importance of metal‐specific responses when interpreting *mt* levels in fish used for environmental monitoring; lastly, it is important to recognize that the in vitro model does not fully replicate in vivo physiology, and that temporal or concentration constraints may have limited the induction of metals such as Pb, which might require longer exposures, alternative concentrations, or different timing to elicit a detectable response.

## Conflicts of Interest

The authors declare no conflicts of interest.

## Supporting information


**Data S1:** Supplementary Information.

**Data S2:** Supplementary Information.

## Data Availability

The data that support the findings of this study are available from the corresponding author upon reasonable request.
