# Supplementary material for: A Multimodal Approach to Identify Metallothionein Metal Inducers in Nile Tilapia: Insights From Molecular Docking and Hepatocyte Exposure
Source: J Appl Toxicol. 2025 Sep 2;46(2):590–600. doi: 10.1002/jat.4914 (PMC12791088; doi:10.1002/jat.4914)
Supplement: Supplementary file 1 — Data S1: Supplementary Information. [file JAT-46-590-s001.docx]

**Supplementary Material 1** – Scores for molecular docking between metals and Zn-binding proteins

| **Code - PredZinc** | **Protein** | **MIB2 result** | **Cu^2+^** | **Mn^2+^** | **Zn^2+^** | **Cd^2+^** | **Hg^2+^** | **Pb^2+^** |
| --- | --- | --- | --- | --- | --- | --- | --- | --- |
| 8128.ENSONIP00000027420 | Arnt2 | 7daca50ea6401771fecc59196b25cee0_ | 6.429 | 3.544 | 8.971 | 5.193 | 2.511 | - |
| 8128.ENSONIP00000043167 | ENSONIP00000043167 | 32fbc989a0bccb6310f6d6eaf1b8d35d_ | - | 1.858 | 4.417 | 1.525 | 2.541 | - |
| 8128.ENSONIP00000049788 | ENSONIP00000049788 | 6fcc911eabb535c9e1425a2844c816bd_ | - | 4.131 | 8.271 | 6.91 | - | 4.935 |
| 8128.ENSONIP00000013074 | I3JW29_ORENI | 3a59d1d8f88aa29ae11dfe76bb0cf84a_ | - | 2.122 | 5.205 | 5.769 | - | 3.936 |
| 8128.ENSONIP00000038758 | I3KBK4_ORENI | 717cfb8b6a60692050c82553f0525c83_ | 4.568 | 1.822 | 6.047 | 4.87 | 7.016 | 4.279 |
| 8128.ENSONIP00000022038 | I3KLP3_ORENI | a6d531d6333920dbe54462a2fc548ab6_ | 3.245 | 3.141 | 6.305 | 3.352 | 4.688 | 2.733 |
| 8128.ENSONIP00000031773 | LOC100691863 | 6b5ef1dd869fe4624107545d9fee68a1_ | - | 6.002 | 5.101 | 2.449 | - | 4.304 |
| 8128.ENSONIP00000000013 | LOC100700393 | 9dc610a6b4e881befccc84a6185c9ddd_ | 3.801 | 2.064 | 5.407 | 3.649 | 8.396 | - |
| 8128.ENSONIP00000058661 | LOC100701476 | fae20713fb08aa79e0755dcb59f870cf_ | 3.108 | - | 4.262 | 3.413 | 5.764 | - |
| 8128.ENSONIP00000074180 | LOC100705328 | 05b4ef93408a470b992eaf2d4d252f7d_ | 7.407 | 2.348 | 7.747 | 4.587 | 7.615 | 3.907 |
| 8128.ENSONIP00000015386 | LOC100707622 | 60980ed0298fe26769af0c68557216a5_ | 4.816 | 3.664 | 9.209 | 3.005 | - | 2.671 |
| 8128.ENSONIP00000008110 | LOC100708914 | b0c12e2a35e84e39e22bc1fa32e64fb6_ | 2.131 | 7.405 | 7.369 | 5.996 | - | 6.942 |
| 8128.ENSONIP00000038385 | LOC102079309 | d534ba4aad0b84054b1f952eb3ba9502_ | 1.892 | 3.253 | 6.1 | 2.793 | - | - |
| 8128.ENSONIP00000024865 | PIP5K1A | 8e958aef248ec1eaa1ce05242d5e2ea1_ | - | 4.557 | 5.78 | 7.44 | - | 3.99 |
| 8128.ENSONIP00000059343 | Pip5k1c | fe0c5f77a5f7a42ec0272cb051625f7e_ | 6.962 | 2.333 | 4.299 | 2.946 | - | 2.745 |
| 8128.ENSONIP00000020837 | SIM1 | 8d831f0d0d59823b08ecbe472d304464_ | 3.271 | 4.453 | 9.244 | 4.697 | 8.072 | 2.445 |
| 8128.ENSONIP00000058598 | TK | 16c5d35f57b76e46cf1544cb9b675793_ | - | 1.676 | 5.455 | 0.93 | 2.473 | 1.821 |
| 8128.ENSONIP00000035314 | TKT | 09bc892d7dc23432147c2c0320eaf0c4_ | 1.97 | 3.323 | 5.078 | 1.05 | 5.665 | 3.459 |
| 8128.ENSONIP00000002302 | actr8 | a10331764a58d33795f3ffbb360e111e_ | 3.142 | 1.454 | 4.283 | 2.719 | - | 2.462 |
| 8128.ENSONIP00000079937 | ahrr | 8e43635c569f63b43916f173590d628d_ | 4.381 | 6.738 | 8.956 | 6.231 | 2.4 | 2.438 |
| 8128.ENSONIP00000041689 | arpc2 | b4048a62d8865342b9be208e50601907_ | 9.336 | 4.331 | 5.355 | 2.873 | 2.794 | 1.948 |
| 8128.ENSONIP00000005081 | bckdha | 7f5d8ef6e051dd5ffb906a05ea22adaf_ | - | 1.866 | 5.235 | 3.304 | 2.797 | 2.806 |
| 8128.ENSONIP00000006994 | cstf3 | 3b2f76ea56a633f1a3dc03fb0811589b_ | 4.119 | 2.015 | 5.932 | 1.785 | - | - |
| 8128.ENSONIP00000001884 | epas1 | 9dd963039041fb4d1c9865e052add0d0_ | 3.567 | 2.639 | 7.333 | 6.96 | 4.156 | 3.142 |
| 8128.ENSONIP00000002225 | esrra | 70f94d8d9bdcc927f61d90c2cfcac2d1_ | - | - | 4.861 | - | - | - |
| 8128.ENSONIP00000033612 | fdft1 | 0b8f506cc2a41229b354946b7771cda7_ | 4.319 | 1.513 | 5.048 | 3.711 | - | 4.942 |
| 8128.ENSONIP00000031774 | hif1a | 1a291ed75ec29830f5c14ac2b6cd313b_ | 5.017 | 1.941 | 5.901 | 3.61 | 3.886 | - |
| 8128.ENSONIP00000020420 | mtf1 | 675f5df15604089483dfaece1a2294fc_ | 6.554 | 2.339 | 6.523 | 3.809 | 4.341 | 3.833 |
| 8128.ENSONIP00000055805 | mysm1 | f4a4c4d76010ae42667c129d8ec5d50c_ | - | 3.427 | 8.026 | 7.134 | - | 4.326 |
| 8128.ENSONIP00000053294 | npas4 | d7e573df4c19428feba4afc4a7c175a7_ | 5.607 | 3.306 | 8.238 | 5.147 | 2.358 | - |
| 8128.ENSONIP00000011054 | parvb | 8bc80c721dead817f8ef64cf3c96489f_ | 2.478 | 3.25 | 5.218 | 4.181 | - | 4.217 |
| 8128.ENSONIP00000012112 | ripk1 | f65493a8f411392a4e317d51a652cbb3_ | - | 3.155 | 6.09 | 1.851 | - | 1.896 |
| 8128.ENSONIP00000063014 | rpl13a | 895a79122b99b672f0c702ce5e75fbd1_ | 3.655 | 3.232 | 4.14 | 7.244 | 6.214 | 3.125 |
| 8128.ENSONIP00000049031 | ruvbl2 | ef0fe6034fa403017f09284a75d93866_ | 7.015 | 2.036 | 6.163 | 5.953 | 2.691 | - |
| 8128.ENSONIP00000015908 | slc31a1 | 50a2ab0fa5b0e8b7f6ad341441c7f468_ | 3.925 | 3.29 | 4.772 | 3.112 | - | - |
| 8128.ENSONIP00000003601 | traf2 | dd8d0a1482b4b81f90f33603aedd7cfd_ | 3.925 | 3.29 | 4.772 | 3.112 | - | - |
| 8128.ENSONIP00000039492 | twf1 | d61aa7a22d341033bcf13f1654027cc3_ | - | - | 3.814 | 1.746 | - | 2.358 |
