# Supplementary material for: A Multimodal Approach to Identify Metallothionein Metal Inducers in Nile Tilapia: Insights From Molecular Docking and Hepatocyte Exposure
Source: J Appl Toxicol. 2025 Sep 2;46(2):590–600. doi: 10.1002/jat.4914 (PMC12791088; doi:10.1002/jat.4914)
Supplement: Supplementary file 2 — Data S2: Supplementary Information. [file JAT-46-590-s002.docx]

**Supplementary Material 2** – Gene expression analysis: N_0_ values calculated using LinRegPCR and fold change

| Sample | Experiment | N0 (mean eff) - no plateau - stat efficiency | Versus control (fold change) |
| --- | --- | --- | --- |
| Control | 1 | 2.80E-02 | 1.00 |
| Control | 2 | 1.43E-02 | 1.00 |
| Control | 3 | 2.14E-02 | 1.00 |
| Control | 4 | 3.35E-02 | 1.00 |
| Control | 5 | 8.23E-02 | 1.00 |
| Control | 6 | 9.01E-02 | 1.00 |
| Cadmium 0.2 mg L⁻¹ | 1 | 2.24E-02 | 0.80 |
| Cadmium 0.2 mg L⁻¹ | 2 | 3.37E-02 | 2.35 |
| Cadmium 0.2 mg L⁻¹ | 3 | 1.49E-02 | 0.70 |
| Cadmium 0.2 mg L⁻¹ | 4 | 6.23E-02 | 1.86 |
| Cadmium 0.2 mg L⁻¹ | 5 | 9.90E-02 | 1.20 |
| Cadmium 0.2 mg L⁻¹ | 6 | 1.11E-01 | 1.23 |
| Cadmium 1 mg L⁻¹ | 1 | 5.01E-02 | 1.78 |
| Cadmium 1 mg L⁻¹ | 2 | 4.75E-02 | 3.31 |
| Cadmium 1 mg L⁻¹ | 3 | 5.35E-02 | 2.51 |
| Cadmium 1 mg L⁻¹ | 4 | 4.94E-02 | 1.48 |
| Cadmium 1 mg L⁻¹ | 5 | 1.61E-01 | 1.95 |
| Cadmium 1 mg L⁻¹ | 6 | 2.89E-01 | 3.21 |

| Sample | Experiment | N0 (mean eff) - no plateau - stat efficiency | Versus control (fold change) |
| --- | --- | --- | --- |
| Control | 1 | 1.37E-01 | 1.00 |
| Control | 2 | 2.01E-01 | 1.00 |
| Control | 3 | 2.89E+00 | 1.00 |
| Control | 4 | 2.08E+00 | 1.00 |
| GnRH-A | 1 | 1.62E-01 | 1.19 |
| GnRH-A | 2 | 2.21E-01 | 1.10 |
| GnRH-A | 3 | 2.82E-01 | 0.10 |
| GnRH-A | 4 | 8.26E-02 | 0.04 |

| Sample | Experiment | N0 (mean eff) - no plateau - stat efficiency | Versus control (fold change) |
| --- | --- | --- | --- |
| Control | 1 | 1.55E-02 | 1.00 |
| Control | 2 | 4.32E-02 | 1.00 |
| Control | 3 | 4.98E-03 | 1.00 |
| Control | 4 | 3.92E-03 | 1.00 |
| Cadmium 2 mg L⁻¹ | 1 | 5.29E-02 | 3.41 |
| Cadmium 2 mg L⁻¹ | 2 | 3.55E-01 | 8.22 |
| Cadmium 2 mg L⁻¹ | 3 | 1.16E-02 | 2.32 |
| Cadmium 2 mg L⁻¹ | 4 | 2.84E-02 | 7.25 |
| Copper 10 mg L⁻¹ | 1 | 1.30E-02 | 0.84 |
| Copper 10 mg L⁻¹ | 2 | 3.49E-02 | 0.81 |
| Copper 10 mg L⁻¹ | 3 | 9.68E-03 | 1.95 |
| Copper 10 mg L⁻¹ | 4 | 1.15E-02 | 2.94 |
| Lead 5 mg L⁻¹ | 1 | 1.21E-02 | 0.78 |
| Lead 5 mg L⁻¹ | 2 | 6.21E-02 | 1.44 |
| Lead 5 mg L⁻¹ | 3 | 1.19E-02 | 2.39 |
| Lead 5 mg L⁻¹ | 4 | 1.12E-02 | 2.86 |
| Manganese 10 mg L⁻¹ | 1 | 1.38E-02 | 0.89 |
| Manganese 10 mg L⁻¹ | 2 | 4.02E-02 | 0.93 |
| Manganese 10 mg L⁻¹ | 3 | 8.28E-03 | 1.66 |
| Manganese 10 mg L⁻¹ | 4 | 1.10E-02 | 2.81 |
| Mercury 0.1 mg L⁻¹ | 1 | 9.27E-03 | 0.60 |
| Mercury 0.1 mg L⁻¹ | 2 | 2.66E-02 | 0.61 |
| Mercury 0.1 mg L⁻¹ | 3 | 7.38E-03 | 1.48 |
| Mercury 0.1 mg L⁻¹ | 4 | 1.35E-02 | 3.46 |
